# Automated data cleaning of paediatric anthropometric data from longitudinal electronic health records: protocol and application to a large patient cohort

**DOI:** 10.1038/s41598-020-66925-7

**Published:** 2020-06-23

**Authors:** Hang T. T. Phan, Florina Borca, David Cable, James Batchelor, Justin H. Davies, Sarah Ennis

**Affiliations:** 1 NIHR Southampton Biomedical Research Centre, University Hospital Southampton, Southampton, UK; 2 University of Southampton, Southampton, UK; 3 University Hospital Southampton NHS Foundation Trust, Southampton, UK

**Keywords:** Endocrinology, Health care, Medical research

## Abstract

‘Big data’ in healthcare encompass measurements collated from multiple sources with various degrees of data quality. These data require quality control assessment to optimise quality for clinical management and for robust large-scale data analysis in healthcare research. Height and weight data represent one of the most abundantly recorded health statistics. The shift to electronic recording of anthropometric measurements in electronic healthcare records, has rapidly inflated the number of measurements. WHO guidelines inform removal of population-based extreme outliers but an absence of tools limits cleaning of longitudinal anthropometric measurements. We developed and optimised a protocol for cleaning paediatric height and weight data that incorporates outlier detection using robust linear regression methodology using a manually curated set of 6,279 patients’ longitudinal measurements. The protocol was then applied to a cohort of 200,000 patient records collected from 60,000 paediatric patients attending a regional teaching hospital in South England. WHO guidelines detected biologically implausible data in <1% of records. Additional error rates of 3% and 0.2% for height and weight respectively were detected using the protocol. Inflated error rates for height measurements were largely due to small but physiologically implausible decreases in height. Lowest error rates were observed when data was measured and digitally recorded by staff routinely required to do so. The protocol successfully automates the parsing of implausible and poor quality height and weight data from a voluminous longitudinal dataset and standardises the quality assessment of data for clinical and research applications.

## Introduction

With the availability of digital electronic health systems, ‘big’ clinical data has become more accessible to the research community^1,2^. The big data era, which includes using data obtained from heterogeneous digital sources, has enabled novel opportunities for conducting empirical clinical research. At the same time there are challenges using such data for research purposes, including the need to adapt existing and develop new methodologies to cope with the scale and complexity of the data^3^. However, a more fundamental issue for researchers is the requirement to undertake data cleaning, as incorrect clinical measurements entered into an electronic health record (EHR) will significantly affect the quality of dataset. Data cleaning can be time-consuming and involve multiple stages including detailed data analysis to identify error types, data inconsistencies, outlier detection and implement data transformation where required^4,5^. Thus, developing automated methods for data cleaning is desirable.

Height and weight are the most commonly recorded anthropometric measures for the assessment of child health in both clinical practice and research studies. Longitudinal height measurements give an indication of well-being and perturbations may be an indication of nutritional, endocrine, cardiac or other abnormalities that should prompt a clinical decision for investigation or intervention. Body mass index (BMI), defined by heights and weights, may be used to establish risks of prevalence of diseases^6^. In children, longitudinal changes of BMI provide insight into predisposition to health problems such as obesity, hypertension, type 2 diabetes and nutritional insufficiency.

World Health Organisation (WHO) guidelines^7^ can be used to exclude biologically implausible values (BIV) from the EHR for childhood height, weight and BMI data, by converting the measurements to standard deviation scores (SDS) and using defined parameters to exclude extreme values (e.g. height to age z-score (HAZ) exclusion if < −6 or >6). However, there are few studies which have evaluated methods for cleaning periodical longitudinal anthropometric data^8^. For example, some have identified BIVs for annual longitudinal values where the mean changes of BMI values exceed 3SDS or −3SDS and height decrements greater than 1 inch/year, and mean increases in height>3SDS^9,10^. Others^10^ have suggested removing weight measurements where annual changes exceed 22.7 kg or 27.2 kg if the individual was severely obese at baseline, any height decrease and any height increase >15 cm a year. These methods were developed for identifying extreme changes in periodical measurements and do not detect less extreme changes and so are not applicable to children where growth is dynamic. Neither are they applicable to the big-data scenario where anthropometric measurements are non-periodical. More recently the jack-knife residual method, applicable to paediatric patients with ≥4 datapoints, was suggested and applied to a paediatric anthropometric dataset for children ≤2 years old^11^. Although simple to use, it can be too strict in defining the range of plausible values hence not allowing more pronounced fluctuations in longitudinal data that are typical in the paediatric clinical setting where an individual can reduce or gain significant weight during or after a treatment period^12,13^.

University Hospital Southampton (UHS) is a large teaching and research hospital serving a population of nearly 3.5 to 4 million people in South Hampshire. The Southampton Children’s Hospital of UHS initiated electronical recording of anthropometric measurements in 2012 and subsequently developed an Electronic Growth Chart (EGC) which was rolled out for use across departments in the hospital in 2013^14^. Since then, anthropometric data on children has been systematically recorded, improving the accuracy of growth data presentation on a growth chart and enhancing the experience of sharing growth data by clinicians between paediatric specialities. It has also presented an opportunity for research studies to use longitudinal routine patient care anthropometric data and make correlations between childhood growth and development of disease or efficacy of therapy. However, data recorded for routine clinical care by end-users can be prone to typographical or default value entry errors often related to time pressure for care delivery. Hence it is necessary that the anthropometric data be cleaned and processed before it is used for research purposes.

In this study, we developed an automated protocol for identifying outliers of longitudinal routine paediatric height and weight measurements using state-of-the-art outlier detection methods. Concurrently, a subset of UHS electronic paediatric height and weight data of patients aged 2–20 years old, the gold-standard dataset manual curated for parameter optimisation, were assessed for data quality. We demonstrate how dataset scrutiny can identify and target training needs in anthropometric assessment in a teaching hospital.

## Materials and methods

### Anthropometric data scope and extraction

Electronically recorded height, weight measurements and date of birth was extracted for all patients admitted to UHS from 1932–2018 where the patient’s age at date of measurement was between 2–20 years. Data prior to 2008 were paper-based archived data transcribed into the electronic EPR system since its introduction in UHS. *Measurements are recorded to an accuracy of 1 decimal place for weight* (*kg) and height (cm)*. The occupation and department of the staff members entering the data was also captured. Measurements of children of age less than 2 years were not considered in this assessment as the absence of gestational age data prevented accurate calculation of height for age z-scores (HAZ), weight for age z-scores (WAZ) and weight for height z-scores (WHZ). From the raw measurements of height (H, metre) and weight (W, kg), BMI was calculated as W/H^2^ and HAZ, WAZ and WHZ were calculated using the LMS method^15^.

### Data quality indicators

In assessing the quality of the captured anthropometric height and weight measurements, established data quality indicators for children ≥2 years of age were applied: (i) standard deviation (SD) of HAZ, WAZ and WHZ^16^ (ii) Myer’s Index (MI) for height and weight where MI is a measurement of digit preference of recorded data^17^. Myer’s Index calculates the divergence in the frequency of the ending digit in the measurements compared with the expected uniform distribution where there is no digit bias. The higher the value, the more biased the measurement towards a digit or two in all measurements, reflecting rounding effects.

### Conventional data cleaning

The thresholds for normal ranges of HAZ, WAZ and WHZ specified by the WHO Child Growth Standards^18^ were applied for height, weight and BMI measurements. Those satisfying the condition of HAZ, WAZ or WHZ being within the [−6,6], [−6,5] and [−5,5] ranges respectively were retained for further analysis.

### Implausible flagging of sparse data

When longitudinal measurement data were sparse e.g. the number of entries per individual was less than four, an implausible increment or decrement flag was applied e.g. gain or loss of >25% of weight within one day; gain or loss of >40% of weight within three months; gain or loss of >50% of weight within one year; gain of >15% of height within three months; any decrease in height exceeding 1 cm were flagged for manual checking.

### Outlier flagging method for longitudinal data

For outlier flagging of longitudinal anthropometric measurements, robust regressions of the linear regression methodology was adopted^19^. Robust regressions can handle multiple outliers by introducing residual statistics including influence measurements such as Cook’s distance, DFFITS, DFBETAS^20^ (see Supplementary for method details). Datapoints with influence statistics exceeding suggested thresholds are temporarily removed from the inference and the regression parameters are re-estimated from the remaining data. This results in a regression line that best fits the most reliable data. It is this regression line that is used to discriminate outlying datapoints from the entire set of datapoints using the SD fold threshold *θ*.

### Additional checks on height data

In addition to robust regression analysis of the data to detect outliers, height measurements were additionally inspected to flag anomalies such as variation in adult height and/or height decrease over time as follow. Final adult height is generally reached at approximately 18 years^21^, therefore, variation >1 cm from the median height measurements of patients older than 18 years flagged an error in data recording. Additionally, any decrease in height exceeding 1 cm also prompted a flag to cross check recorded data manually. This check was applied regardless of the number of datapoints in any set of measurements.

Details of the overall longitudinal height and weight data outlier flagging protocol is summarised in Box 1.

Box 1 Summary of final protocol for outlier flagging for longitudinal height and weight measurements of a patient

1. Flag data not satisfying WHO guidelines for heights, weights and BMIs whose SDS values fall beyond the ranges [−6,6], [−6,5] and [−5,5] respectively, remain n datapoints
2. If n < 4: assess the implausible increments/decrements of height and weight measurements:
  i. For weight: for each pair of consecutive measurements, use the following method to flag extreme changes as below:
    Time span ≤ 1 day: beyond ±25%
    Time span ≤ 3 months: beyond ± 40%
    Time span ≤ 1 year: beyond ± 50%
  ii. For height
    If time span ≤ 3 months, height increase is ≥15%
    If height measurement at time point is at least 1 cm smaller than time point, flag data at time point.
3. With the remaining data, where n > =4:
  - Apply the ordinary least square (OLS) linear regression method of the SDS values as a linear function of age (number of variables k = 1)
  - Calculate influence values: Cook’s distance, dffits, dfbeta for age. Retain data that have Cook’s distance <1, |dffits | <2 and | dfbeta_age | <2/ to re-estimate the regression line and obtain the SD of the residuals.
  - Any patient whose SD of the residuals for height or weight larger than 0.47 or 0.76 respectively has their whole series of measurements flagged for manual inspection.
  - Where the SD of the residuals for height or weight is ≤1, flag any individual datapoint with residual error exceeding *θ* x SD where *θ* is 2.9 for weight and 2 for height (as informed by parameter tuning).
  - For height data:
    i. Perform adult height check: for age measurements not flagged in (2c) within the range 18–20 years, calculate median value for that individual M_h_, and flag as outlier any height measurement difference exceeding 1 cm.
    ii. Across all age ranges and for data not already flagged, perform height decrease check. If height measurement at time point is at least 1 cm smaller than time point, flag data at time point.
4. If the total number of datapoints flagged (by any step) exceed 40% of the longitudinal data, the whole series of longitudinal data is flagged for manual inspection.

### Parameter tuning

Typically, datapoints exceeding 2 times the SD (*θ*) of any series of measurements are nominally flagged as outliers, corresponding to an outlier rate of 5%^22^. However, for voluminous datasets of growth data in children, this parameter may be unnecessarily stringent. The tuning of *θ* was facilitated by a ‘gold-standard’ dataset from UHS, manually curated by an endocrinologist (JHD), where each patient had ≥7 datapoints (Supplementary text). This gold-standard dataset consisted of 6,279 patients with 89,258 weight measurements and 4,396 patients with 55,688 height measurements. Of these, 208 (0.23%) weight and 302 (0.54%) measurements were deemed ‘implausible’ by the endocrinologist. Additional height checks identified a further 191 (0.34%) height measurements failing the adult height check and 1,237 (2.22%) flagged by the height decrease check, totalling 1,730 flagged height measurements (3.11%). This yielded a gold-standard dataset with a defined set of ‘true’ errors.

Sensitivity and specificity metrics were evaluated for *θ* ∈ [1.5,5.5] using the gold standard dataset. Here, a true positive (TP) was defined as a datapoint identified as an outlier that was deemed clinically implausible by the clinician, a true negative (TN) was a value that was not flagged as an outlier by our method and identified as plausible by the clinician, a false positive (FP) was a true plausible value wrongly flagged as an outlier, and a false negative (FN) was a truly implausible value not flagged as an outlier by the protocol. Therefore, the positive predictive value (PPV) is an important metric to consider. Ideally, any given protocol should maximise the number of true outliers as a proportion of all data flagged for manual review while maintaining good sensitivity to detect all true outliers.

The gold-standard UHS data were used to calculate sensitivity and PPV for *θ* ∈ [1.5,5.5] (Fig. S4). For both height and weight, it was desirable to maintain sensitivity above 0.9 while maximising the PPV. Hence for height, the typical value of *θ* = 2 was selected but for weight measurements, it was observed that increasing *θ* to 2.9 maintained sensitivity above 0.9 but had a dramatic effect on reducing the manual curation of false positive outliers (Table 1). These values were used in the final protocol described in Box 1.

**Table 1:** Contingency tables for chosen values of *θ* for weight and height and their sensitivity and PPV^#^.

*(a) Contingency table of weight outlier flagging*					*(b)* Contingency table of height outlier flagging				
Weight *θ* = 2.9		Manual curation by clinician			Height *θ* = 2		Manual curation by clinician		
		Impossible	Plausible				Impossible	Plausible	
Flagging by protocol	Outlier	189	2,110	2,299	Flagging by protocol	Outlier	1,694	2,775	4,469
	Plausible	19	86,940	86,959		Plausible	36	51,183	51,219
		208	89,050	89,258			1,730	53,958	55,688
Sensitivity = 90.87%					Sensitivity = 97.91%				
PPV = 8.22%					PPV = 37.91%				

^#^PPV is Positive Predicted Value, defined as the proportion of positive results that are true positive, PPV = TP/(TP + FP).

The final selected values of *θ* were applied to gold standard data sets for height and weight respectively. From 55,688 height measurements, a subset of 4469 measurements (representing 2635 patients) were flagged as outliers for manual inspection. Approximately 92% of the data passed checks and could be automatically classified as plausible. Of the 8% of flagged measurements, the 1237 (2.2%) due to decreases in height may be excluded without further clinical review and only 5.8% of the data may be subjected to further expert review or excluded depending on application. Importantly, the protocol failed to flag 36 measurements across 25 patients that the clinician subsequently flagged as implausible. This represented 0.06% of possible erroneous measurements that would go undiscovered by automated cleaning. Similarly, for weight, 2299 (2.6%) measurements from 1875 patients were flagged as requiring manual expert review while 97.4% of the data passed automated checks. Only nineteen datapoints (0.02%) that were deemed by the clinician as implausible were missed by the protocol.

All the data processing and protocol implementation was performed using the open-source programming language Python version 3.7^23^. The ordinary least square method OLS from the Python package statsmodel^24^ was used to perform LR. The script for calculating SDS values of anthropometric measurements and outlier detection described by the pipeline is available for use from https://github.com/hangphan/peanof/. This includes the portable Docker container^25^ where all dependencies required for running the script were set up and ready to be executed on any environment where Docker is made available.

### Ethics and information governance

The study was approved by the IG management team of the University Hospital of Southampton (UHS). Ethics approval from the Research Ethics Committee and Health Research Authority, and informed consent was waived by the internal review board at the R&D Department of UHS as this is a combination of an Audit against WHO guidance and Service Evaluation. The anthropometric data in UHS were retrospective data and anonymised. All methods used in this study were performed in accordance with the relevant guidelines and regulations.

## Results

### Data quality of gold-standard longitudinal data

**Figure 1:**
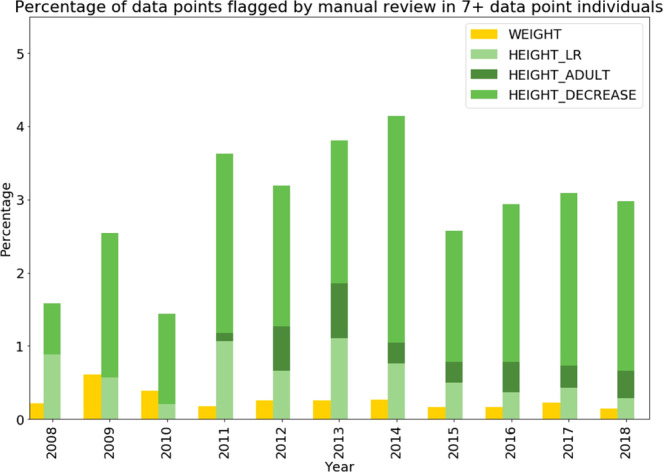
Percentage of datapoints identified as true errors in the gold standard dataset stratified by year for weight and height, weight for height. Outliers were split into three types: height outlier flagging using linear regression (LR), height entry error with adult height check and height with height decrease check.

The ‘gold-standard’ UHS height and weight dataset enabled assessment of true data quality. Chronologically, both height and weight measurements across the 2008–2018 were stable with an error rate of ~3% for height and 0.2% for weight (Fig. 1). The discrepancy in error rates between the two measurements was largely attributable to decreases in height which were deemed physiologically impossible.

**Figure 2:**
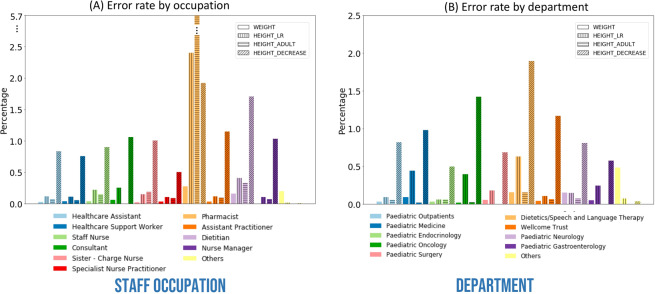
Manual outlier curation results of UHS gold standard paediatric height and weight data: (**a**) Percentage of outliers for each of the occupation categories for weight, height using LR, height with adult height check, and height with height decrease check. (**b**) Percentage of outliers for each of the department categories for weight, height using LR, height with adult height check, and height with height decrease check.

Outlier rate by occupation was highest in the Pharmacist group (0.27%) followed by Others (0.20%) and Dietician (0.16%) for weight. The Pharmacist group recorded the most errors in height as assessed through manual review (2.4%) and using the adult height check (5.7%, Fig. 2a). This likely reflects the pharmacist’s focus on estimated weight and not height for prescribing purposes.

By department, the Others group has the highest error rate for weight (0.48%) followed by Dietetics/Speech and Language Therapy and Paediatric Neurology (0.16%, Fig. 2b). For height data, the highest rate of data deemed implausible though manual review was observed in Dietetics/Speech and Language Therapy (0.63%) followed by Paediatric Medicine (0.44%) and Paediatric Oncology (0.40%). Additional height checks saw the highest combined error rate in Dietetics/Speech and Language Therapy (2.05%) followed by Paediatric Oncology (1.25%, Fig. 2b*)*.

### Application of automated cleaning protocol to the entire UHS paediatric height and weight dataset (n = 68,595 patients)

#### UHS data summary and characteristics

**Figure 3:**
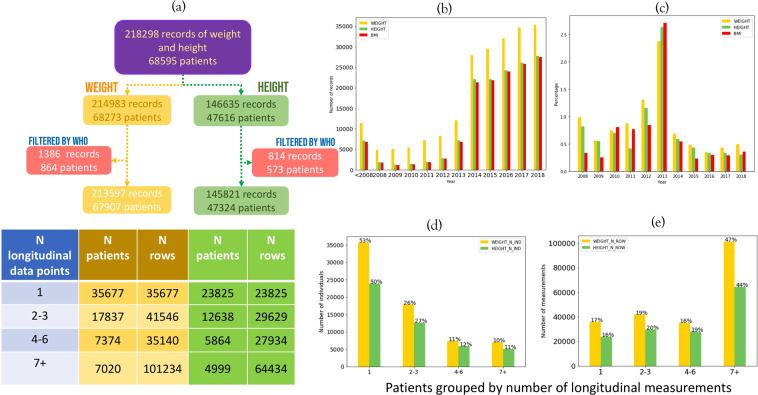
UHS age 2–20 years’ height and weight data (1932–2018) summary: (**a**) Number of patients and records of height and weight, broken down by number of datapoints per patients. (**b**) Total number of height, weight and BMI measurements over time from prior to 2008 to 2018 (**c**) Percentage of data flagged by WHO guidelines over time. (**d**) Number of patients within groups of patients defined by their number of longitudinal datapoints for height and weight. (**e**) Number of height and weight records per group of patients binned by number of datapoints per patient.

The entire cohort contained all records for patients aged 2–20 years, dating from 1932 to 31/12/2018. A total of 214,983 weight measurements (68,273 patients) and 146,635 height measurements (47,616 patients) were obtained for 68,595 paediatric patients in the UHS EPR (Fig. 3a), resulting in 142,643 BMI values (46,479 patients).

The number of records was low prior to 2008 (1932–2008) and increased from 2008, reflecting the gradual introduction of EPR system into UHS departments, with a sharp increase in 2014 when the EGC was introduced at the end of 2013 (Fig. 3b). The number of weight measurements recorded was about 30% higher than that of height during 2014–2018 period. Additional description regarding age group at initial measurement, length of follow-up time is presented in Supplementary (Fig. S4a,b).

Patients were grouped by their respective number of longitudinal height and weight measurements. There is an excess of patients with a single measurement entry and these represent approximately half of the cohort, reflecting paediatric patients with a single hospital visit to departments such as emergency. Patients with ≥7 entries for height and weight represented ~10% of the cohort but contributed almost half of the entire dataset for both height and weight (Fig. 3d,e). These represent the patient population whose ill health may confer growth and developmental irregularities requiring frequent monitoring.

#### Data quality by conventional quality indicators

The number of records failing WHO child growth standard guidelines for weight, height and BMI measurements were 1,386 (0.95%) and 814 (0.38%) and 677 (0.47%) respectively. The percentage of records excluded based on WHO limits was highest in 2013 at 2.37%, 2.64%, and 2.71 for weight, height and BMI respectively (Fig. 3c). This coincides with the gradual introduction of EGC into various departments across UHS in 2013, reflecting a transient increase in error rate during the transition period to the electronic recording of data. A comparison of the five years preceding the transition to electronic data recording and the five years following 2013 identified a significant reduction (p_weight_ = 9.97 × 10^−23^, p_height_ = 1.05 × 10^−8^) in these extreme data recording errors.

The SD of HAZ, WAZ and WHZ was calculated and compared against reported ranges of SD observed in the 52-country DHS survey^16^ (Table 2). The SD values prior to exclusion of WHO extreme datapoints fell significantly outside the expected ranges. However, after exclusions of these extreme values, the observed SD values for height, weight and BMI z-scores fall within the expected limits.

**Table 2:** Standard deviation of WAZ, HAZ and WHZ of the UHS 2–20 anthropometric measurement data.

	WAZ	HAZ	WHZ
DHS RANGE OF SD	1.01–1.49	1.08–2.33	1.01–2.02
PRE-WHO PROCESSING SD	5.29	5.90	15.55
POST-WHO PROCESSING SD	1.45	1.32	1.36

**Figure 4:**
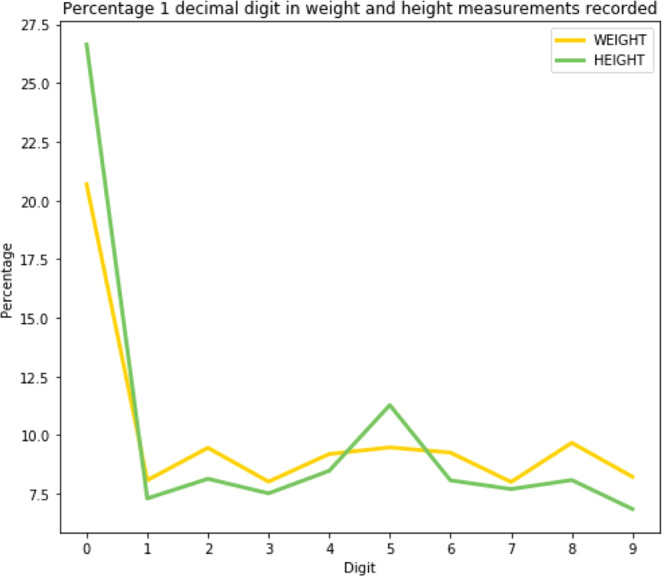
One decimal place digit distribution for height and weight measurements, demonstrating the bias in recording height and weight measurements, rounding to the precision of kg for weight and the precision of cm or 0.5 cm for height. This bias is reflected in the Myers’ index of height and weight measurements.

The Myer’s Index (MI) for digit preference of height data (excluding WHO extreme values) is consistent with the average observed across 51 countries in the DHS survey (MI_UHS_ = 17.91, MI_51_country_average_ = 17.8, Fig. 4). The MI for weight data is higher (MI_UHS_ = 10.69, MI_51_country_average_ = 4.6) suggesting a greater tendency for estimation in UHS weight data.

#### Data quality indicators by occupation and department of entry staff

The quality of the extracted data was also scrutinised by staff occupation and department to understand the most likely source of erroneous data and target the training in anthropometric assessments.

**Figure 5:**
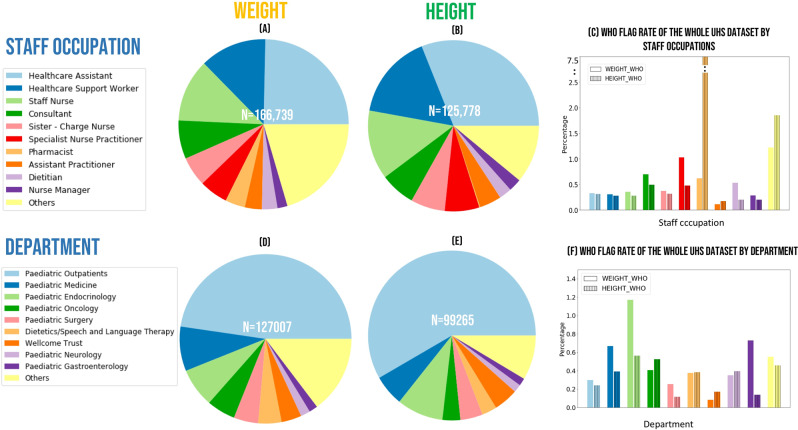
UHS data characterisation by occupation and by department of staff entering the data (**a**) Weight records by occupation (**b**) Height records by occupation (**c**) Percentage of height and weight data flagged by WHO rules by occupation (**d**) Weight records by department (**e**) Height records by department (**f**) Percentage of height and weight data flagged by WHO rules by department.

For 75% of the observed data, the occupation and department of the staff member entering the data was available for evaluation. Ninety-three different staff occupations across 96 different departments were noted and the ten staff occupations that most frequently entered height and weight measurements are presented in Fig. 5a,b. Healthcare assistants most frequently recorded weight and height data (24% and 30% respectively) followed by Healthcare support workers, Staff nurses and Consultants.

Application of the WHO flags for extreme values identified a low and consistent level of less than 1% of likely data entry error across occupations (Fig. 5c). The most striking peak in this type of error was 7.5% noted in the height data entered by pharmacists. However, given pharmacists entered only a very small proportion of the overall height data (n = 214 records) this higher error rate reflects a very small number (n = 16) extreme values.

The Paediatric outpatient department contributed most data for weight and height measurements (47% and 58% respectively; Fig. 5d,e). The WHO violation rate by department was small and relatively consistent across departments. The highest rate identified was 1.2% amongst weight values recorded within the Paediatric Endocrinology *department (*Fig. 5f*)*.

#### Outlier detection for patients with longitudinal records in UHS dataset

For those with 2–3 height measurements, the implausible flagging method identified 655 (2.21%, 607 patients) height decreases >1 cm (Table 3). No height increases>15% within 3 months were detected. For those with 2–3 weight measurements, the extreme weight change check resulted in 119 (0.29%, 114 patients) measurements flagged. For patients with ≥7 datapoints, the protocol Section 3c flagged 1,303 weight measurements from 71 patients whose weight data had a large residual SD (>0.76) and protocol Section 3d identified 2,573 weight measurements (2,055 patients) as LR outliers.

**Table 3:** Number of flagged implausible values by the application of the final outlier flagging protocol to the UHS 2–20 data set from 1932 to 31/12/2018.

Patient group	Filter	Weight	Height
All	WHO	1,386 (n = 864)	814 (n = 527)
2–3	Extreme change	119 (n = 114)	655 (n = 607)
4–6	OLS robust, few remain	680 (n = 170)	292 (n = 73)
	Large SD	114 (n = 24)	296 (n = 61)
	LR	3,626 (n = 3,531)	3,029 (n = 2,987)
	Adult height	N/A	114 (n = 77)
	Height decrease	N/A	357 (n = 365)
≥7	OLS robust, few remain	0	0
	Large SD	1,303 (n = 71)	699 (n = 46)
	LR	2,573 (n = 2,055)	3,412 (n = 2,581)
	Adult height	N/A	222 (n = 121)
	Height decrease	N/A	1,395 (n = 674)

Similarly, Section 3c of the protocol flagged 699 height measurements (46 patients) as having large SD and Section 3d identified 3,412 height measurements (2,581 patients) as LR outliers and the additional adult height checks (Section 3e) flagged a further 1,617 datapoints (Table 3).

## Discussion

This study presents a bespoke protocol for automated anthropometric data cleaning that has been tested across a sizeable dataset captured from a regional teaching hospital in South England. While more than half the patients represented within the dataset had only a single measurement recorded, approximately half of the collected data are from a small proportion (~10%) of the patients with multiple longitudinal records. This subset is likely to be enriched for patient cohorts that are the subject of health research.

The WHO parameters to detect BIVs was a good first pass analysis to detect overtly incorrect data. WHO outliers represented <1% of recorded measurements. The discrepancy in data quality indicator before and after WHO guideline BIV detection is a strong indication of a small subset of extreme data entry errors (e.g. records of 1 kg or 100 cm and/or transposition errors in weight and height measurements respectively). It is essential these conspicuous errors are automatically flagged and excluded from the data before any model fitting approaches are applied. Additionally, the WHO thresholds should be applied with caution in specific clinical settings where patients with true clinical data may fall outside these parameters (e.g. growth disorders, morbid obesity) but still have BIVs longitudinally.

All protocols for cleaning data are limited when data are sparse. We have developed a robust regression framework for automatic identification of erroneous data that performs most reliably across data series with at least seven measurements. Application of the protocol automatically classifies the majority of records as plausible values, leaving a small proportion of flagged data that can be either discarded or manually reviewed. The protocol is computationally cheap to implement and provides assurance of minimal and consistent quality standard for downstream analysis. The availability of the code in Github and Docker communities allows for the protocol can be adopted easily in different settings. The protocol is also modifiable, particularly in the arbitrary thresholds chosen for height and weight checks to reflect less extreme changes in non-clinical settings.

Encouragingly, overall error rates in paediatric data were low, with transient fluxes in data quality observed over periods where new systems were implemented. A higher error rate was noted in measurements of height compared to weight, largely due to very small decreases in height likely resultant from inconsistencies in measuring techniques, e.g. shoes on or off. The error profile by occupation demonstrated that staff routinely required to measure and enter the data tended to record better quality measurements.

The application of the protocol allows an assessment and rapid feedback regarding data quality in EHR systems which is valuable for identifying and targeting training needs and data entry practice. This will further contribute to the overall impact of improving the quality of data available for longitudinal clinical assessment and patient management as well as enhancing input data quality for large-scale digital healthcare research.

## Supplementary information

Supplementary information.

## Data Availability

The data that support the findings of this study are available from UHS, but restrictions apply to the availability of these data, which were used under license for the current study, and so are not publicly available. Data are however available from the authors upon reasonable request and with permission of UHS.
