# Supplementary material for: Automated data cleaning of paediatric anthropometric data from longitudinal electronic health records: protocol and application to a large patient cohort
Source: Sci Rep. 2020 Jun 23;10:10164. doi: 10.1038/s41598-020-66925-7 (PMC7311482; doi:10.1038/s41598-020-66925-7)
Supplement: Supplementary file 1 — Supplementary information. [file 41598_2020_66925_MOESM1_ESM.pdf]

Supplementary:

Automated data cleaning of paediatric height and weight data from longitudinal electronic health records: protocol and application to a large patient cohort

Hang T.T. Phan[1,2*], Florina Borca[2,3], David Cable[3], James Batchelor[1,2], Justin H. Davies[3#], Sarah Ennis[1,2#]

Affiliation

[1] NIHR Southampton Biomedical Research Centre, University Hospital Southampton, Southampton, UK

[2] University of Southampton, Southampton, UK

[3] University Hospital Southampton NHS Foundation Trust, Southampton, UK

[#]These authors contributed equally to the preparation of this manuscript

[*] Corresponding author

1. Longitudinal data outlier detection protocol development

It is desirable to expedite the cleaning and curation of contemporary electronic healthcare data. A linear relationship between age and the height for age z-scores (HAZ) and weight for age z-scores (WAZ) is expected[1]. It is therefore possible to exploit this expected relationship using linear regression (LR) methodology to automate the detection of a minimal subset of outlier data that is flagged for manual curation. Reducing the burden of curation to a minimal set facilitates discrimination of erroneous data entry from clinically plausible measurements.

Using LR outlier detection methodology, a regression line is firstly fitted to the observed data by minimising a target function, usually the mean of squared errors where the error is the difference between the observed value and that predicted from the regression analysis. Outliers can then be flagged if the error exceeds certain threshold - often twice the value of the standard deviation (SD) of the errors.

However, substantial outliers can pose a serious problem to any standard LR method as they can skew the original line fitting [2]. This issue cannot be surmounted with very sparse data but can be addressed where there are sufficient datapoints using the jack-knife method [1]. This leave-one-out LR method iteratively removes a single data, fits the model on the remaining data and evaluates the excluded datapoint against the fitted model. This approach is more sensitive in detecting singleton outliers compared to the standard residual LR method [3], however the method loses power if multiple outliers exist within the data [2]. However, the jack-knife approach is computationally expensive, scaling quadratically with the number of datapoints.

To this end, the robust regression methods [2] was adopted in our outlier detection method development as it is robust with multiple outliers by using influence measurements such as Cook's distance[4], DFFITS, DFBETAS. Cook's distance estimates the influence of a datapoint when performing least square LR analysis [4]. It measures the effect of removing a given observation to the LR analysis. Datapoints with large Cook's distance (>1) reflect large residuals (difference between predicted and true value) or high leverage. Studentized DFFITS measures the influence of a single datapoint in LR analysis. It is calculated as the change in the predicted value of a point when it is excluded from the LR divided by the estimated SD of the model at that point [5]. It was suggested that datapoints with DFFITS $>2\sqrt{\frac{k}{n}}$ should be further investigated where k is the number of parameters in the LR model[4]. Using height and weight data, age is the only feature to make prediction of HAZ or WAZ, hence $k=1$. DFBETAS measures the difference in any given parameter estimate with and without individual datapoints [6]. A datapoint with DFBETAS $> 2\sqrt{\frac{1}{n}}$ is suggested for further investigation.

Datapoints with influence statistics exceeding suggested thresholds are temporarily removed from the inference and the regression parameters are re-estimated from the remaining data. This results in a regression line that best fits the most reliable data without the computational expense of the jack-knife approach. It is this regression line that is used to discriminate outlying datapoints from the entire set of datapoints using the SD fold threshold θ.

2. Number of datapoints for effective outlier detection using linear regression (LR) methodology

Using real data from a single patient's WAZ values, we performed a simulation on the number of datapoints from which linear regression-based method becomes effective. The simulation is from a series of WAZ values from an arbitrarily selected patient with 27 measurements (a_i, s_i) (i=1…27), where a_i is age and s_i is WAZ value at age a_i. The process of a single simulation is as followed:

1. For n ranges from 4 to 12, randomly select n datapoints from 27 datapoints and place them in a subset which is a list of n pairs of values (a_{nj}, s_{nj}) (j=1..n)

2. For each sampled subset of size n:

   For j in 1..n:

   a. Replace s_{nj} with $y \in [-6, 6]$ (incrementing by 0.1)
   b. Perform the OLS LR analysis to detect outliers as described in the main text.
   c. Plot the simulated value y on the graph depending on where it is in the series. If the value y is not flagged, it is plotted in blue, and if it is flagged, it is plotted in yellow

The simulation was implemented in Python. The pseudo-code written in Python format is available in Figure S1. The simulation process was repeated four times (Figure S2). The pattern of flagged and not flagged data across the range of values of y at different values of n, demonstrates the ability of the LR model to distinguish outliers in the series. It is clear from the simulation results that the LR method accumulates power to distinguish possible outliers in series with at least seven datapoints, visible by the yellow bands and the top and bottom of each datapoint in the simulated series.

```python
originalVec = [[a_i, s_i]] # original vector of (age, measurement), size of vector = 27

for n in range(4,12):
    simVec = random(n, originalVec) # create a vector of n random pairs of datapoints from originalVec
    for j in range(1,n):
        y = -6
        originalValue = simVec[j][1]
        while y <=6:
            simVec[j][1] = y
            flags = outlierDetection(simVec) # run the outlierDetection method based on updated simVec

            # plot the simulated point on the age-measurement plot, colored by the flag value
            # flag = True means the simulated point was judged as an outlier by the algorithm
            # flag = False means the simulated point was judged as plausible by the algorithm

            if flags[j] == True:
                scatterPlot(simVec[j][0], simVec[j][1], color = 'Yellow')
            else:
                scatterPlot(simVec[j][0], simVec[j][1], color = 'Blue')
            y += 0.1
        simVec[j][1] = originalValue #restore the original value at index j to move on to simulation at j+1
        # color the original point as red on the graph
        scatterPlot(simVec[j][0], simVec[j][1], color = 'Red')
```

Figure S 1. Python-based pseudo-code for the simulation to estimate the best number of datapoints for linear regression of height and weight z-score measurements.

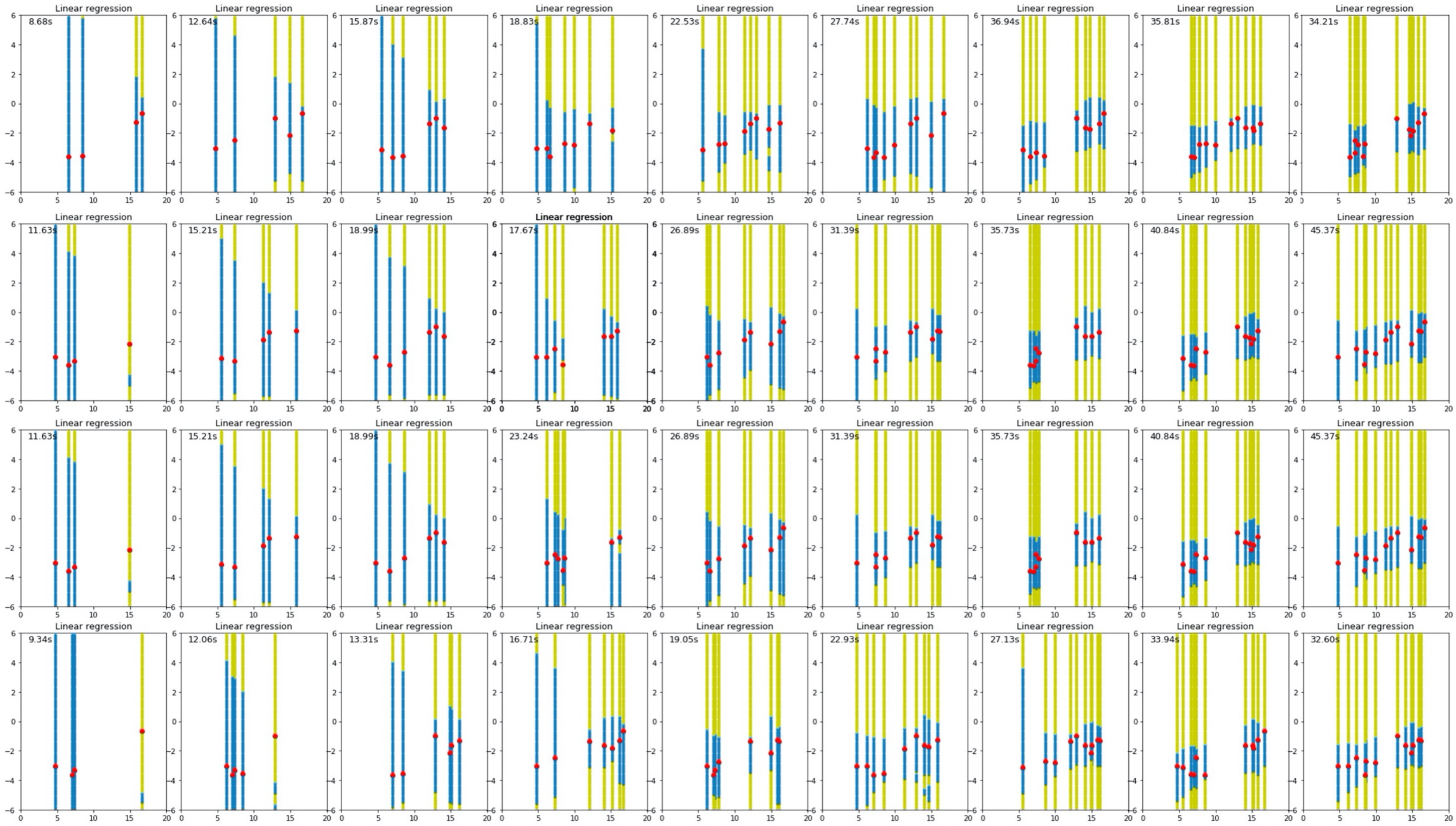
Linear regression
8.68s
12.64s
15.87s
18.83s
22.53s
27.74s
36.94s
35.81s
34.21s
11.63s
15.21s
18.99s
17.67s
26.89s
31.39s
35.73s
40.84s
45.37s
11.63s
15.21s
18.99s
23.24s
26.89s
31.39s
35.73s
40.84s
45.37s
9.34s
12.06s
13.31s
16.71s
19.05s
22.93s
27.13s
33.94s
32.60s

Figure S2. Simulation results to assess the number of datapoints from which OLS linear regression becomes effective in discriminating outliers. The figure shows simulation results of four independent runs of the process: (i) Given a real data set of weight measurements of a patient in UHS with 27 measurements, for each run, with n ranging from 4 to 12, a set of n datapoints is randomly selected from the given set. (ii) Then for each datapoints in the sampled set, replace WAZ value at this point by a value ranging from -6 to 6 incremented by 0.1, and perform the OLS LR method on these n datapoints (iii) The replaced point is marked in blue if the LR method accepts that point as 'Plausible', in yellow if 'Implausible', and the original datapoint is marked in red. The original datapoints are not necessarily an indicator of a true non-outlier, and merely reflect possible real-life data. For the value of n from 4 to 6, the LR method exhibits a wide acceptance range and it is only with more datapoints that the acceptance range is reduced, indicating discriminatory power for outlier detection where there are seven or more datapoint.

3. Parameter tuning and evaluation

Typically, individual datapoints exceeding $\theta = 2$ times the standard deviation of any series of measurements are nominally identified as outliers, corresponding to an outlier rate of 5% [7]. However, for voluminous datasets of growth data in children, this parameter may be unnecessarily stringent and invoke a higher rate of manual data inspection than is necessary. We tuned θ to identify the optimal value of this parameter that minimised the set of data for manual inspection while having high probability of flagging truly erroneous measurements for scrutiny. This was achieved by ranging θ within a wide possible range and evaluate the sensitivity and specificity of each value of θ. A truth set or gold standard data set was required to facilitate this parameter tuning.

As with all regression modelling, the method had greater robustness to detect outliers as the number of longitudinal measurements increased and reliably identified outliers with ≥7 measurements (Supplementary Figure S2). The gold standard subset was defined as that containing all data collected up to 1 July, 2018 and included only patients with ≥7 measurements within WHO parameters for each of height and weight respectively. These data were reviewed manually by a clinician (JHD) to provide expert opinion on the clinical plausibility of recorded measurements. For all patients in this set, each height or weight measurement was classified as either 'plausible' or 'implausible' by the clinician by visual inspection of the patient's growth chart and scatter plot of HAZ or WAZ and by additional height checks.

The gold standard dataset was further restricted to those with *SD* of the LR residuals within the 99th percentile (Figure S3). The final gold standard subset included 6,279 and 4,396 patients totalling 89,258 and 55,688 weight and height measurements. Of these, 208 (0.23%) weight and 302 (0.54%) measurements were deemed 'implausible' by the endocrinologist. Additional height checks identified a further 191 (0.34%) height measurements failing the adult height check and 1237 (2.22%) flagged by the height decrease check, totalling 1,730 flagged height measurements (3.11%).

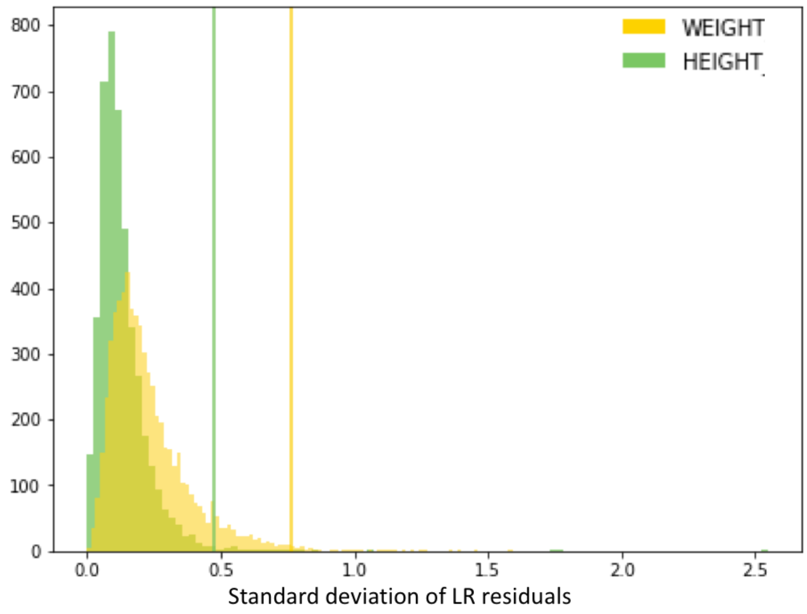

Figure S3. Distribution of standard deviation of HAZ and WAZ residuals in OLS linear regression for every patient with ≥7 datapoint. The distributions are presented with 99 percentile vertical lines for height and weight (green and yellow respectively). Patients with SD measurements beyond the 99 percentile of the distribution for height and weight respectively are flagged for manual curation.

Parameter tuning results

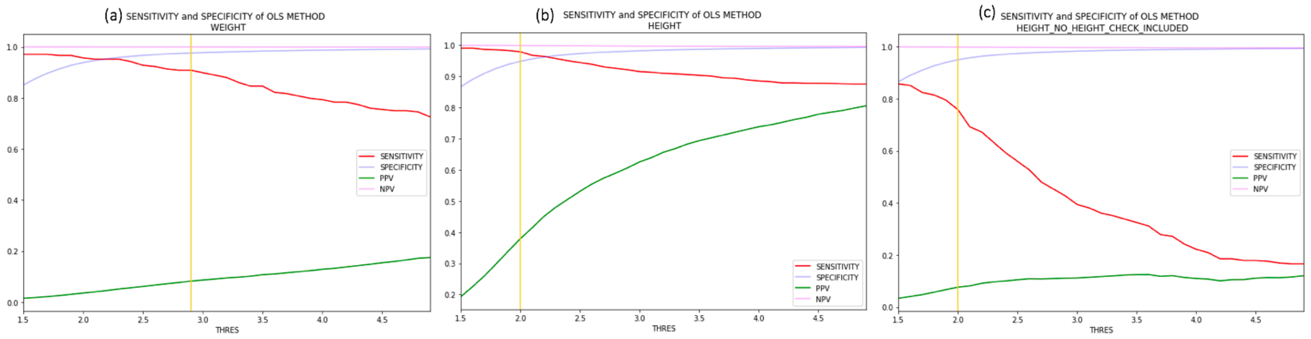

Figure S4. Parameter tuning of robust linear regression in outlier flagging protocol development. Each subfigure demonstrates the change of sensitivity, specificity, positive predictive value (PPV) and negative predictive value (NPV) with the SD fold threshold θ (1.5-5.5). (a) Evaluation metrics for weight outlier flagging protocol (b) Evaluation metrics for height outlier flagging protocol (c) Evaluation metrics for height outlier flagging protocol without additional height checks. Specificity and PPV values are consistently high (>75% and >99%) with the increase of θ and provide little discriminatory power to the models. The tuning therefore relies on the assessment of sensitivity and NPV to inform the optimal compromise between sensitivity to detect outliers and the burden of time required for expert manual review of flagged cases (NPV). Vertical yellow lines represented the selected values of θ that maintain a balance between sensitivity (>90%) and manual curation work requirement (NPV).

4. **UHS data - additional description**

Figure S5 demonstrate the distributions of patients by age at first measurement and length of follow-up time for height and weight data in the UHS EPR system for patients aged 2-20 years.

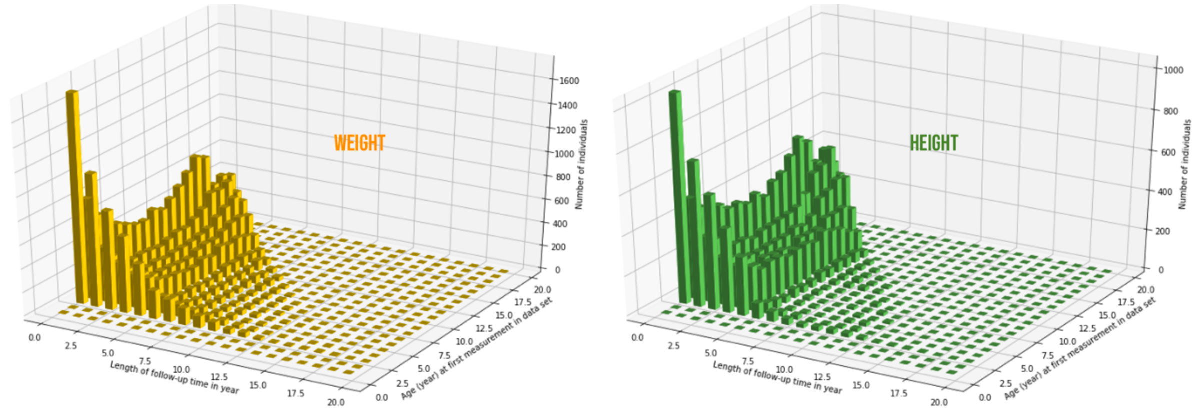

Figure S5. Histogram of number of patients with the age when they first had the measurement in the data set and the length of follow-up time in years for weight and height

References

1. Shi J, Korsiak J and Roth DE. New approach for the identification of implausible values and outliers in longitudinal childhood anthropometric data. *Ann Epidemiol* 2018; 28: 204-211 e203. 2018/02/06. DOI: 10.1016/j.annepidem.2018.01.007.
2. Rousseeuw PJ and Leroy AM. *Robust regression and outlier detection*. John Wiley \& Sons, Inc., 1987, p.329.
3. ATKINSON AC. Two graphical displays for outlying and influential observations in regression. *Biometrika* 1981; 68: 13-20. DOI: 10.1093/biomet/68.1.13.
4. Cook RD. Detection of Influential Observation in Linear Regression. *Technometrics* 1977; 19: 15-18. DOI: 10.1080/00401706.1977.10489493.
5. Belsley DA, Kuh, E., Welsch, R. E. . Detecting Influential Observations and Outliers. *Regression Diagnostics*. New York: John Wiley & Sons, 1980, pp.11-16.
6. Peter K. Robust Estimation. *A Guide to Econometrics (5th edition)*. Cambridge The MIT Press, 2003, pp.372–388.
7. Seo S. *A Review and Comparison of Methods for Detecting Outliers in Univariate Data Sets*. University of Pittsburg, 2006.
